# COVID-19: Crisis Management in Congenital Heart Surgery

**DOI:** 10.1177/2150135120931398

**Published:** 2020-06-04

**Authors:** Elizabeth H. Stephens, Joseph A. Dearani, Kristine J. Guleserian, David M. Overman, James S. Tweddell, Carl L. Backer, Jennifer C. Romano, Emile Bacha

**Affiliations:** 1 Department of Cardiovascular Surgery, Mayo Clinic, Rochester, Minnesota; 2 Department of Congenital Heart Surgery, Medical City Children’s Hospital, Dallas, Texas; 3 Division of Cardiovascular Surgery, Children’s Minnesota, Minneapolis, Minnesota; 4 Division of Cardiothoracic Surgery, Department of Surgery, Cincinnati Children’s Hospital Medical Center and the University of Cincinnati, Cincinnati, Ohio; 5 Division of Cardiovascular-Thoracic Surgery, Ann & Robert H. Lurie Children’s Hospital, Chicago, Illinois; 6 Department of Cardiac Surgery, CS Mott Children’s Hospital, University of Michigan, Ann Arbor, Michigan; 7 Division of Cardiothoracic Surgery, Department of Surgery, Columbia University Irving Medical Center/NewYork-Presbyterian Hospital, New York, New York

## Preface

Our nation’s health care infrastructure faces unprecedented challenges in the face of the COVID-19 pandemic, and the congenital heart disease (CHD) community is no exception. These challenges include looming resource scarcities of equipment, personnel, and blood. In addition, there are the substantial infection risks to patients, family members, and staff. These factors necessitate thoughtful but often difficult decisions on how to best triage patients with CHD. Our relatively small workforce adds another dimension to the challenge, since the rapid spread of COVID-19 could result in program closure at a moment’s notice secondary to insufficient personnel from infection or quarantine. Although many sectors of our society can be placed on hiatus during this period of crisis, our patients’ diseases continue to require care, particularly among newborns and infants who often necessitate operations during a narrow temporal window for satisfactory outcomes.

Practitioners are tasked with optimizing care in the presence of the current and rapidly changing circumstances. Although statements have been published relative to adults undergoing operations,^1^ guidance with respect to CHD patients is currently lacking and is the goal of this document. As we have seen in other countries facing this pandemic, the thoughtful allocation of resources is paramount to the overall welfare of the community^2,3^ and has led to such strategies in the United States.^4,5^ The safety of our patients, health care providers, and our communities is our chief concern.

This document is not meant to be a guideline but is designed to provide *guidance* for decision-making as we face unparalleled challenges related to congenital cardiac surgery care during this pandemic. The circumstances are rapidly changing, even hourly; therefore, the principles outlined here are meant to be fluid and adaptable. They should be continually reappraised in the context of the dynamic circumstances within a given institution, population base, and geographic location. These principles can also provide a framework for prioritization of operations in other situations when there is lack of resources or personnel, or both. This document is not meant to be prescriptive, but rather serves as an outline of guiding principles to be interpreted in a particular context. Lastly, while individual anomalies may be specified, each patient should be considered individually in the context of their clinical status, disease state, institution, and community.

Perhaps more than ever, these times require us as a specialty, albeit small but with a valuable scope of skill sets, to *collaborate and cooperate*. This includes the sharing of knowledge and resources, patient transfer in selected situations, and communicating frequently among ourselves to provide emotional support and mental fortitude during these periods of stress and isolation.

Congenital heart surgeons have a track record of effectively working together to advance the specialty, promote quality training and education of our surgical community, and through bold innovation solve some of the most difficult clinical challenges. We are now confronted with a different kind of challenge—a public health care crisis. We can rise to meet this challenge by using our collaborative and intellectual abilities to problem solve, plan, and prevail for the benefit of children and adults suffering from CHD. We must work together with strong leadership from our institutions and central, shared decision-making teams to handle the influx of issues and concerns related to the pandemic.

## Triage of Surgical Patients

In the setting of substantially limited resources, proper prioritization of patients requiring operations is of paramount importance. To date, the biggest difficulties have not been COVID-19 infections in CHD patients, which have been rare. The biggest threat to our patients is the sudden lack of resources, including the requisition of operating rooms (transformed into intensive care units), ventilators, and health care providers for the fight against COVID-19. As a result, the surgical schedule must be culled to only the most urgent cases, which in our specialty is very difficult with many gray zones. Many factors pertaining to an individual case must be weighed, including (1) resource utilization, such as anticipated ventilator duration, intensive care unit stay, blood product use, and other supplies that are or may become limited; (2) clinical status of the patient and risk of delaying operations; (3) risk of exposure for the patient, family, and health care staff; (4) comorbidities and complexity of the procedure with implications on the use of hospital resources; (5) training in teaching hospitals may have to be curtailed and the most experienced surgeons used liberally; and (6) the safety of the patient’s social and clinical situation if the operation is delayed. These decisions will often be made in the context of a hospital that is weighing the needs of other patients from different specialties with similar needs.

The timing of an operation is determined by a variety of factors, including the clinical status of the patient, recommendations of the Centers for Disease Control and Prevention, government officials, and professional societies (eg, American College of Surgeons), and local hospital protocols. Importantly, this is a shared decision-making with the patient, family, and other specialists with transparency regarding the risks and benefits of proceeding with the operation vs waiting. The difficulty of this decision-making regarding timing is compounded by the realization that the duration of this pandemic and thus the delay for a given patient remains unknown.

Table 1 is provided as *general guidance* regarding the timing of intervention for various diagnoses, depending on the patient’s clinical status and other factors. It is not exhaustive and not meant to be a guideline, but highlights common conditions and scenarios. As stated earlier, this should be interpreted with flexibility depending on the current state of resources within a given institution and community. It is important to note that ongoing, close cooperation and communication between the surgical and medical teams is essential to ensure an accurate diagnosis and effective treatment strategy with the fewest resources and involved personnel. In the setting of unrestricted resources, certain conditions can be managed in a standard fashion, but consideration during this time should be given to alternative approaches that may drain fewer resources or result in shorter hospitalization, or both; for example, a ductal stent may optimize resource utilization for a given institution as opposed to a surgical systemic-to-pulmonary artery shunt. Depending on the impact of this pandemic, there may be circumstances in the near future when it may not be appropriate or feasible to offer interventions to high-risk patients requiring high resource utilization with anticipated poor outcomes.

**Table 1.** Congenital Lesions and Surgical Prioritization^a^

Patient	**Emergent** (24-48 Hours of Diagnosis When Adequate Resources)	**Urgent** (Within 1-2 Weeks When Adequate Resources)	**High Priority Elective** (>2 Weeks When Adequate Resources)
**Neonate**	*Note*: *Timing for categories will depend on resources available*, *institutional protocols*, *and other pending cases*		
Shunts, mixing lesions			
TAPVC/cor triatriatum	Obstructed	Increasing gradient	
TGA		<1 week if IVS	2-4 weeks if VSD
Truncus arteriosus			If stable
Tetralogy of Fallot	Severe hypoxemia/hypercyanotic spells	Symptomatic	
Regurgitant lesions			
Ebstein anomaly		Refractory medical mgmt	
Obstructive lesions			
Coarctation	Shock unable to stabilize on PGE	If able to stabilize on PGE	
Critical aortic stenosis	Shock unable to stabilize on PGE	If able to stabilize on PGE	
PGE-dependent pulmonary blood flow			
PA/IVS		If PDA stent not available	
PGE-dependent systemic blood flow			
HLHS	Intact, restrictive atrial septum if BAS not available	Case and surgeon dependent	Case and surgeon dependent
Other			
Shunt	Shunt thrombosis	Shunt stenosis	
Arrhythmias	Symptomatic congenital heart block unable to medically manage/externally pace		
ALCAPA	Once medically stabilized		
**Infant**			
Shunts left → right			
VSD		Symptomatic CHF on medical mgmt	Failure to thrive
Shunts right → left			
Tetralogy of Fallot		Symptomatic (spells, cyanosis) on medical mgmt	
Regurgitant lesions			
AVSD			Trisomy 21 with pulmonary overcirculation, consider age of patient to optimize repair, significant regurgitation unable to manage medically
Ebstein anomaly			Increasing right-sided heart failure on medical mgmt
Mitral regurgitation			Symptomatic CHF on medical mgmt
Aortic regurgitation	Acute, hemodynamically unstable		Enlarging LV, decreasing LV EF, symptoms
Obstructive lesions			
Valve prosthesis	Thrombosed prosthesis		
AS/LVOTO			Decreasing LV EF, symptoms
RVOTO			Decreased RV function
Other			
Shunt	Shunt thrombosis	Shunt stenosis	
DCM/HF		CHF failing medical mgmt	Failure to thrive
BDCPA candidate			Increasing cyanosis with current shunt, shunt stenosis
**Children**			
Regurgitant lesions			
Mitral regurgitation			Symptomatic CHF on medical mgmt
Aortic regurgitation	Acute, hemodynamically unstable		Enlarging LV, decreasing function, symptoms
Obstructive lesions			
AS/LVOTO			Decreasing LV function, symptoms
Valve prosthesis	Thrombosed prosthesis		
RV-PA conduit obstruction	Severe stenosis with severe RV dysfunction and/or ventricular arrhythmias	Severe stenosis with RV dysfunction and/or systemic RV pressure	Worsening right-sided failure
Other			
DCM/HF		CHF failing medical mgmt	Failure to thrive
Fontan candidate			Increasing cyanosis
Endocarditis	Cardiogenic or septic shock despite max medical mgmt	Hemodynamically stable, but uncontrolled infection	Per guidelines
AAOCA	Recent cardiac arrest, hemodynamically unstable, on mechanical support	History of aborted sudden death, chest pain with minimal exertion	
Combined lesions (ie, MR and subAS)	Hemodynamic compromise	Moderate/severe individual lesions	
**Adult Congenital**			
Regurgitant lesions			
Ebstein/TR			Increasing right-sided heart failure on medical mgmt
Mitral regurgitation			Symptomatic CHF on medical mgmt
Aortic regurgitation	Acute, hemodynamically unstable		Enlarging LV, decreasing LV EF, symptoms
Obstructive lesions			
HCM		Syncope/presyncope	
Aortic stenosis			Decreasing LV EF, symptoms
RV-PA conduit obstruction	Severe stenosis with severe RV dysfunction and/or ventricular arrhythmias	Severe stenosis with RV dysfunction and/or systemic RV pressure	Worsening right-sided failure
Other			
Endocarditis	Cardiogenic or septic shock despite max medical mgmt	Hemodynamically stable, but uncontrolled infection	Per guidelines
AAOCA	Recent cardiac arrest, hemodynamically unstable, on mechanical support	History of aborted sudden death	

^a^ Lesions are listed under the age group(s) in which they most commonly present. Not included in this table is orthotopic heart transplantation. The decision to accept a donor heart during this pandemic depends on the recipient’s clinical status, the estimated risk of the donor’s potential exposure to COVID-19 in their community and hospital, and the prevalence of COVID-19 in the hospital and community of the recipient in light of the immunosuppression the recipient will receive.

AAOCA, anomalous aortic origin of the coronary arteries; ALCAPA, anomalous left coronary artery from pulmonary artery; AS/LVOTO, aortic stenosis/left ventricular outflow tract obstruction; AVSD, atrioventricular septal defect; BAS, balloon atrial septostomy; BDCPA, bidirectional cavopulmonary anastomosis; CHF, congestive heart failure; DCM/HF, dilated cardiomyopathy/heart failure; EF, ejection fraction; HCM, hypertrophic cardiomyopathy; HLHS, hypoplastic left heart syndrome; IVS, intact ventricular septum; LV, left ventricle; mgmt, management; MR, mitral regurgitation; PA/IVS, pulmonary atresia/intact ventricular septum; PDA, patent ductus arteriosus; PGE, prostaglandin-E1; RV, right ventricle; RVOTO, right ventricular outflow tract obstruction; subAS, subaortic stenosis; TAPVC, total anomalous pulmonary venous connection; TGA, transposition of the great arteries; TR, tricuspid regurgitation; VSD, ventricular septal defect.

## Preserving the Workforce and Regional Collaboration

Given the small size of each institutional workforce practicing congenital cardiac surgery (median of 3 surgeons per practice in the United States),^6^ which possesses unique skill sets not replaceable by other providers, strategies to maintain the integrity of the workforce are crucial. Institutions may redeploy members of the congenital cardiac surgery team to other patient care settings, further depleting resources and also increasing exposure. In response, using workplace strategies to reduce overall exposure, such as 1-week on/1-week off rotation schedule for selected health care providers, appropriate use of personal protection equipment (PPE), and downsizing of clinical teams and worksites, seems prudent.

Vigilant surveillance for symptoms among team members is critical given the close proximity of congenital cardiac team members working together: 1 exposed or positive provider can put the entire team and program at risk because of quarantine requirements, resulting in a program being shut down because of insufficient staff to provide care. Precautions should also be taken for nonclinical support staff to minimize their exposure. Strategies of remote teleworking and rotational schedules should be applied to all staff when feasible.

Regional *collaboration* should be considered, with the realization that small programs are at risk for closing in the setting of infected staff members or those requiring quarantine. While neighboring congenital cardiac surgery programs may have had a history of competition, the importance and necessity of cooperation and collaboration cannot be overemphasized during this time of public health care crisis. *A patient-centric mindset should be the overarching emphasis*, and programs and practitioners should find ways to work together and support each other. Discussions could include strategies for reciprocal hospital privileging that allow staff to move from one site to the other if needed, sharing COVID-19–related perioperative protocols or other newly created institutional care strategies, and crisis management guidelines that respond to the changing needs of COVID-19. In addition, critically ill patients at institutions facing severely depleted resources may be best served by transferring to institutions with more plentiful resources.

## Surgical Consultation and Clinic Visits

Babies will continue to be born each day, and the 1% incidence of CHD will remain unchanged during this time of crisis. Evaluation and early operations for many children must continue while much of the world is on hold. An adaptive clinical model should be applied to minimize exposure and prioritize patients for operations during this crisis period. Differentiating who requires surgical evaluation for potential intervention during this time and who does not can be accomplished with a careful history, virtual visits, and review of diagnostic laboratory tests and imaging. After a multidisciplinary discussion, further imaging or laboratory work can be obtained as needed to allow for accurate risk stratification and surgical planning.

## COVID-19 Exposure and Social Distancing

An ongoing concern is the relative risk of hospital exposure to COVID-19. Although each hospital strives to carefully monitor and appropriately care for COVID-19 patients, in-house patients and health care providers may be positive before symptom onset or knowledge of their status, putting incoming surgical patients and their families at risk. Furthermore, many of our patients’ family members may be residing in group facilities, for example, Ronald McDonald House, also placing them at risk for exposure. The magnitude of such potential risk is unknown, but it must be weighed against the urgency of the operation. COVID-19 has had the biggest clinical impact on older patients, especially those with predisposing conditions; however, younger patients without medical conditions are being affected and have also died. This finding is an important consideration because the congenital cardiac population often has underlying pathophysiological abnormalities and comorbidities that place them at higher risk in the setting of a respiratory illness.

Social distancing can be particularly challenging for our patients, given their age and family structure. Many of our patients have multiple siblings, each with other potential exposures, and depending on their age, require supervision or care by adults. Adequate psychosocial support for the child and their family must also be considered, while being mindful of the risk of COVID-19 exposure and spread. Individual decision-making must be considered in this setting, taking into account specific hospital policies and community recommendations so exposure is minimized; for example, 1 parent bedside during the hospital stay as opposed to additional family members. The adverse psychosocial impact of such a strategy cannot be underestimated, and alternative support mechanisms be provided.

Discussions continue regarding the appropriate use of PPE for health care workers and screening of incoming patients to prevent spread of COVID-19 within the hospital setting. Such policies will inevitably continue to change with PPE supply, evolution of testing, and knowledge regarding the spread of the virus.

Ideally, preoperative COVID-19 testing during this pandemic should be performed on every patient, even asymptomatic patients, as well as their parents. While the testing yield is poor in asymptomatic patients early in the disease before viral shedding,^7^ a positive test would substantially change both decision-making regarding their operations as well as precautions taken during their hospitalization, use of limited PPE, and interaction with providers and family members. How to manage a COVID-19–positive patient with CHD who requires an urgent operation remains unclear because the effects of cardiopulmonary bypass and mechanical ventilation on an asymptomatic or symptomatic COVID-19–positive patient are currently unknown.

## Role of Congenital Cardiac Surgeons in the Care of COVID-19–Positive Noncardiac Patients

Although our priority and focus has centered on patients with CHD, congenital cardiac surgeons also play an integral role in the care of COVID-19–positive patients without cardiac disease. This primarily includes patients with respiratory failure from COVID-19 who may require extracorporeal membrane oxygenation (ECMO) support. ECMO has been infrequently needed for COVID-19–positive children thus far, but the age range of COVID-19–affected patients continues to evolve, with an increasing number of younger patients being affected. Institution of pediatric ECMO is a unique skill that is most often implemented by congenital cardiac surgeons. While participation of the surgeon is often mostly technical (ie, cannulation), it also includes patient selection guidelines, strategies for ECMO application with the fewest personnel, transportation protocols, post-ECMO limb monitoring, and chest washouts, and others. Early consultation with the surgical service and coordinated efforts with clear communication between the involved personnel—surgery, critical care, perfusion, and nursing, and others—cannot be overemphasized because the potential for controversy and disagreement is more likely in the COVID-19 setting. Strategies to limit health care personnel exposure during the institution of ECMO and the care of COVID-19 patients on ECMO also need to be used.

The decision on whether to use ECMO for COVID-19–positive patients can be controversial and will depend on resource availability, comorbidities, and pre-illness clinical status. ECMO should be considered in a pediatric patient who is positive and otherwise healthy before COVID infection in an institution with adequate resources and infrastructure.

## Training and Education

Surgical trainees in the field of CHD are also severely impacted by the current pandemic. Residents and fellows are frequently the frontline for many urgent in-patient emergencies; for example, cardiac arrest in postcardiac surgery patients, ECMO cannulation, placement of central venous or arterial monitoring catheters, and intubation, among others. They are also on a specific and very short timeline for acquiring surgical experience with case number and procedural requirements. However, this must be balanced with the risk of their exposure and well-being. Hospital policies may limit their participation in in-patient activities, and some may require periods of quarantine.

The cumulative effect of these issues could result in a markedly reduced training experience, thus compromising their ability to fulfill certification metrics. Most congenital cardiac surgery residents have already completed 5 to 9 years of elite thoracic surgical training in order to spend 1 to 2 precious years of training at one of the few congenital residency programs in the country. Although postgraduate medical and surgical education has been complemented by various remote or on-line learning modules over the last decade, the bulk of *surgical* learning continues to be centered in the operating room and intensive care unit setting—this experience is essential and not replaceable with on-line education. Some flexibility during this crisis will be considered by the Accreditation Council for Graduate Medical Education and American Board of Thoracic Surgery, but there may be circumstances where extension of the duration of training is needed; Governing Boards and Program Director input will be necessary to ensure competence of the graduating trainee. In the meantime, efforts need to be made to minimize unnecessary exposure to infection, take appropriate precautions, and maximize all supplemental educational measures (eg, video conferencing, virtual classrooms, etc) so trainees do not fall too far behind.

## Mental Toughness and Emotional Stability

The history of our specialty is defined by courage, resiliency, and tenacity. Congenital heart surgeons complete one of the longest and most rigorous training regimens in medicine. It is a specialty marked by long and technically difficult operations. It is a specialty of high risk and high reward, and the cardiac surgical personality thrives on it. Difficult decision making and critical conversations with colleagues and family members are part of our daily experience. As leaders of our teams, we manage pressure and work to address and minimize our team’s fatigue, anxiety, or moral distress. These attributes have never been at a higher premium than at the present moment.

During this pandemic, the difficult decision-making has shifted to an unprecedented realm—who gets operations and when, how limited resources will be used, and how our team will be allocated toward the overall good. With our depth of experience and reservoir of emotional and mental toughness, we as a community are a critical public health resource. Our patients, families, institutions, and regions are depending on us to help them navigate these difficult circumstances. This historic challenge calls on us to, as we have throughout our history, meet the toughest of problems head-on.

We are a small community with a rich network of relationships. We are now trying to find ways to *work together and uplift one another while under duress*. While isolated physically, we are virtually connected, and this support is vital to our success. Frequent and intentional phone calls, sharing personal experiences, and reaching out to colleagues if institutional capabilities are threatened, as well as finding other ways to balance personal and professional life, are necessary to overcome the present challenge.

## Conclusion

Unprecedented times call for unprecedented measures. Prioritization and appropriate timing of operations are necessary at this time. Practical guidance strategies range from ensuring safety and tactics for specific lesions of the patients to maintaining emotional stability of the staff. Our specialty has been marked by solidarity and camaraderie and carries a history notable for collaboration, flexibility, adaptation, and instant readiness. The time to execute these qualities is here and now.
